# Intermediate CD14^++^CD16^+^ monocytes decline after transcatheter aortic valve replacement and correlate with functional capacity and left ventricular systolic function

**DOI:** 10.1371/journal.pone.0183670

**Published:** 2017-08-22

**Authors:** Jonas Neuser, Paolo Galuppo, Daniela Fraccarollo, Jens Willig, Tibor Kempf, Dominik Berliner, Johann Bauersachs, Julian Daniel Widder

**Affiliations:** Department of Cardiology and Angiology, Hannover Medical School, Hannover, Germany; Klinikum Region Hannover GmbH, GERMANY

## Abstract

**Background:**

Transcatheter aortic valve replacement (TAVR) is the method of choice for patients with severe aortic valve stenosis, who are ineligible or at high risk for surgery. Though TAVR leads to a significant reduction in mortality, a notable amount of patients are re-hospitalized early after TAVR. Parameters or biomarkers predicting outcome are therefore needed to identify patients who benefit most. Specific monocyte subsets have been associated with cardiovascular diseases and were shown to possess prognostic value.

**Methods:**

Peripheral blood was drawn before and after transfemoral TAVR with the self-expanding CoreValve, Boston Lotus or the balloon-expanding Edwards Sapien prosthesis. Classical (CD14^++^CD16^−^), intermediate (CD14^++^CD16^+^) and non-classical (CD14^+^CD16^++^) monocyte subsets were determined by flow cytometry. Transthoracic echocardiography was performed before, early after as well as 3 months after the TAVR procedure.

**Results:**

No significant differences in the absolute monocyte counts were found after TAVR. A significant decline in the intermediate monocyte population was though observed early after TAVR (pre 4.01±0.38%, post 2.803±0.34%, *p≤0*.*05*). Creatinine levels stayed stable after TAVR procedure and intermediate monocytes were associated with worse renal function. Monocyte decline was not related to changes in CRP-, noradrenaline, cortisol or aldosterone-levels. The amount of intermediate monocytes correlated with worse cardiac function and predicted the possibility to reach an improvement in NYHA functional class at 3 months after TAVR.

**Conclusions:**

A significant decline of intermediate monocytes occurs shortly after TAVR. High levels of intermediate monocytes were associated with worse cardiac function and predicted poor functional capacity, hinting at a possible prognostic value.

## Introduction

Transcatheter aortic valve replacement (TAVR) has become the method of choice for patients with severe aortic valve stenosis who are ineligible or at high perioperative risk for conventional aortic valve replacement.[1–5] Even though, TAVR significantly improves symptoms and reduces mortality, a relevant amount of patients are re-hospitalized early after valve implantation.[6–10] For perioperative risk assessment, scores like the STS risk score or EuroSCORE II are used. However, not all comorbidities are reflected in these scores and characteristics such as frailty are not considered. Moreover, these risk scores assess surgical risk and were shown to be imprecise predictors of outcome after TAVR.[11] Different biomarkers such as B-type natriuretic peptide or growth differentiation factor-15 have been reported to predict outcome after TAVR as well, and were suggested to improve patient selection.[12–14] However, additional parameters or biomarkers predicting outcome after TAVR are needed to identify patients who benefit most.

Altered distributions of monocyte subsets are associated with various cardiovascular diseases such as coronary artery disease, stroke and atrial fibrillation.[15–18] Human monocyte subpopulations are defined by different expression profiles of the cell surface molecules CD 14 (lipopolysaccharide (LPS) receptor) and CD16 (Fcγ-III receptor).[19] Three subpopulations can be distinguished, classical (CD14^++^CD16^−^), non-classical (CD14^+^CD16^++^) and intermediate (CD14^++^CD16^+^).[20, 21]

CD14^+^ monocytes seems to be increased in patients with severe AS compared with controls and Fingerle-Rowson et al. reported a possible association of high numbers of CD14^+^CD16^+^ monocytes with the clinical condition of critically ill cardiac valve surgery patients.[22, 23] Even though high levels of intermediate monocytes have been shown to possess prognostic value in various cardiovascular disease, knowledge about the role of monocyte subsets in the setting of AS, is rare.[15–17, 24] Beyond that, monocyte subset regulation mechanisms in general and specifically in the condition of AS remain widely unknown. Thus we sought to investigate early changes in circulating monocyte subsets in patients undergoing transfemoral TAVR.

## Materials and methods

All patients gave written informed consent to participate in this study. The study procedures were in accordance with the ethical guidelines of the 1975 declaration of Helsinki and the local ethic committee of the Hannover Medical School approved the study protocol (1894–2013).

We studied consecutive patients (n = 57) with severe AS undergoing elective transfemoral TAVR with the self-expanding Medtronic CoreValve^®^ (n = 10) and CoreValve^®^ Evolut^™^ R (n = 7) or the balloon-expanding Edwards Sapien XT (n = 1), Sapien S3 (n = 36) and Boston Lotus (n = 3) prosthesis at our department between May 2014 and November 2015. Patients undergoing TAVR procedure represent a heterogeneous patient cohort. By taking left ventricular (LV) systolic function, transvalvular gradient and stroke volume into account, different subgroups can be distinguished: Patients presenting with normal LV systolic function and high transvalvular pressure gradient (Ejection fraction (EF) ≥40%, mean gradient ≥40 mmHg) were considered as “classical AS” (n = 45), whereas patients with poor systolic LV function and low pressure gradient (EF <40%, mean gradient <40mmHg) were regarded as “low-flow/low-gradient AS” (n = 6). Beyond that patients with normal EF but low transvalvular pressure gradient (EF ≥40%, mean gradient <40mg) were accounted as “paradoxical low-flow/low-gradient AS”, if stroke volume index was measured <35ml/m^2^ (n = 6).[25]

Patient characteristics concerning general traits, comorbidities and laboratory values were obtained from medical records. Following the TAVR procedure patients routinely were monitored on our intensive care unit for at least 24 hours and received dual anti-platelet therapy (DAPT) consisting out of aspirin and clopidogrel, if no indication for anticoagulation was present. Preexisting co-medication was continued, so far no preexisting or new contraindication existed. Transthoracic echocardiography was performed using Philips EPIQ7 or iE33 ultrasound machines before and between day 4 to 7 after the TAVR procedure as well as 3 months after TAVR during regular follow-up in the out-patient clinic. Parameters such as transvalvular velocities, gradients, left ventricular stroke volume and ejection fraction by using the biplane Simpson’s rule were measured. LVEF and NYHA class were chosen as clinical endpoints. LV function is a predictor for the outcome in aortic valve surgery.[26] NYHA class gives information about the functional outcome of the patients by representing the functional capacity of the patient’s entire cardiovascular system.

Peripheral blood was drawn on day 0 before and once again early after TAVR (day 4 to 7). Monocyte subsets were determined by flow cytometry from blood samples taken either by venipuncture or from a central venous line and transferred immediately on ice to the lab and prepared directly without time delay for monocytes analysis by FACS. Absolute monocyte counts were determined from blood count analyses by an automated hematology analyzer (XT-2000i, Sysmex). Whole blood (100 μl) was incubated with antibodies for 20 minutes at room temperature in the dark. Versalyse Lysing Solution^®^ (Beckman Coulter) was used to lyse red blood cells. After pre-selection (side scatter and forward scatter) monocytes were identified as HLA-DR+ and classified according to CD14 and CD16 expression. Data were acquired on a GalliosTM flow cytometer and analyzed with GalliosTM software (Beckman Coulter). The following antibodies were used: anti-CD14-APC-H7 (BD Biosciences, 561384), anti HLA-DR-FITC (Biolegend, 307604); anti-CD16 eVolve^™^ 605 (eBioscience, 83-0168-42). Detailed gating strategy for identification of monocyte subsets is shown in S1 Fig.

The sympathetic immune system is capable to influence the innate immune system through activation of adrenoceptors.[27–29] To investigate a possible relationship we measured norepinephrine levels by high-performance liquid chromatography with electrochemical detection. Since corticosteroids were shown to have an impact on monocyte subset distribution and basal monocyte function as well, plasma levels of aldosterone and cortisol were determined by ELISA (Hölzel Diagnostika, Köln, Germany).[30]

Values are presented as mean ± SEM and median. Shapiro-Wilk test was used to verify normality of data (S1 Table). Comparison between groups was performed by Mann-Whitney *U* test or *t*-test as appropriate. Statistical analysis was performed with the software StatView version 5.0.1 (SAS Institute Corp.) and R: A language and environment for statistical computing (R Foundation for Statistical Computing, Vienna, Austria. URL https://www.R-project.org/). Multinomial logistic regression was performed by the function *multinom* from R package *nnet*, defining predictors the continuous variable intermediate monocyte (%) and the categorical variable NHYA-Class pre TAVI and outcome, the categorical variable NYHA-Class three months after TAVI. Correlations of intermediate monocytes with cortisol, aldosterone and noradrenaline were determined by the function *chart*.*Correlation* from the R package *PerformanceAnalytics*. Other correlations were evaluated by simple linear regression. Values of *P*<0.05 were considered statistically significant.

## Results

General traits, comorbidities and selected medication of patients are shown in Table 1. Blood count, monocyte subtype counts and selected other laboratory values are presented in Table 2. At baseline all but two patients presented with symptoms of heart failure (≥NYHA II). From the two remaining patients one suffered from recurrent vertigo and in the other case previous cardiac decompensation had occurred. During the follow-up period of 3 months three patients died. One because of norovirus sepsis, one because of renal failure refusing dialyses and the third one, a 97-year-old woman was found dead in her bed by a nurse in the rehabilitation clinic three weeks after TAVR-procedure.

**Table 1:** General traits, comorbidities and functional parameters before TAVR.

**General Characteristics**		
Age	[y]	83.30 ± 0.79 (range 66–97)
Male	[%]	47.37
BMI	[kg/m^2^]	27.14 ± 0.53
NYHA functional class > II pre TAVR	[%]	96.49
ICU stay	[d]	2.44 ± 0.26
Hospital stay	[d]	8.79 ± 0.51
STS Score		5.97 ± 0.39
EUROScore II		6.71 ± 0.65
**Comorbidities**		
CAD	[%]	64.91
Atrial Fibrillation	[%]	52.63
Diabetes mellitus	[%]	26.32
COPD	[%]	14.04
**Functional Parameters**		
Cardiac Index (thermo-dilution/Fick)	[l/min/m^2^]	2.78 ± 0.05 / 2.77 ±0.07
PAsys	[mmHg]	45.10 ± 1.82
AV Gradient max (pre/post/3Mo)	[mmHg]	73.49±2.7 / 22.95 ± 1.39 / 23.06 ± 1.53
AV Gradient mean (pre/post/3Mo)	[mmHg]	44.66 ± 1.64 / 12.91 ± 0.91 / 15.42 ± 2.62
AV VTI	[cm]	107.7 ± 2.77 / 47.38 ± 1.57 / 51.71 ± 1.85
Relevant Mitral Regurgitation^1^ (pre/3Mo)	[%]	28.07 / 29.27
**Medication**		
Anticoagulation	[%]	35.09
Platelet Inhibitor	[%]	56.14
Beta-Blocker	[%]	68.43
ACE-I / ARB	[%]	82.46
Diuretics	[%]	80.70
Calciumcanal-Blockers	[%]	40.35
Statin	[%]	66.67

^1^ Mitral regurgitation was considered relevant, if it was categorized at least moderate. *BMI*—Body Mass Index; *ICU*—Intensive Care Unit; *CAD*—Coronary Arteries Disease; *COPD*—Chronic Obstructive Lung Disease; *PAsys*—Systolic Pulmonary Artery Pressure; *AV*—Aortic Valve, *VTI*—Velocity Time Integral; *ACE-I*—ACE-Inhibitor; *ARB*—Angiotensin-Receptor-Blocker; Data are given as mean ± SEM or as proportion of all cases. Two patients were lost to follow-up. Eight patients did not attend the out-patient clinic for follow-up, however could be reached by telephone interview.

**Table 2:** Blood count and selected other laboratory values.

**Cell counts**		
Leukocytes (pre/post)	[n/μl]	7.14 ± 0.25 / 7.27 ± 0.27
Neutrophiles (pre/post)	[n/μl]	4.58 ± 0.21 / 4.81 ± 0.25
Lymphocyteses (pre/post)	[n/μl]	1.55 ± 0.09 / 1.37 ± 0.07*
Monocytes (pre/post)	[n/μl]	0.03 / 0.79 ± 0.04
• Classical (CD14^++^CD16^−^)	[%] [n/μl]	82.09 ± 0.84 / 84.31 ± 0.91 0.64 ± 0.03 / 0.68 ± 0.04
• Intermediate (CD14^++^CD16^+^)	[%] [n/μl]	4.01 ± 0.38 / 2.80 + 0.34* 0.03 ± 0.003 / 0.02 ± 0.003*
• Non-Classical (CD14^+^CD16^++^)	[%] [n/μl]	8.18 ± 0.55 / 7.47 ± 0.55* 0.06± 0.005 / 0.06 ± 0.005
**Serum analysis**		
Creatinine (pre/post)	[μmol/l]	106.02 ± 5.27 / 102.77 ± 5.10
CRP (pre/post/max)	[mg/l]	6.36 ± 1.37 / 43.54 ± 5.35* / 82.58 ± 7.39
Cortisol (pre/post)	[ng/ml]	184.01 ± 8.43 / 190.58 ± 9.73
Aldosterone (pre/post)	[pg/ml]	110.78 ± 14.08 / 88.96 ± 10.56*
Noradrenalin (pre/post)	[pg/ml]	699.49 ± 113.53 / 657.29 ± 79.66

Blood count and selected other laboratory values obtained before and after TAVR. All data are given as mean ± SEM;

* p<0,05 pre vs. post TAVR.

**Fig 1:**
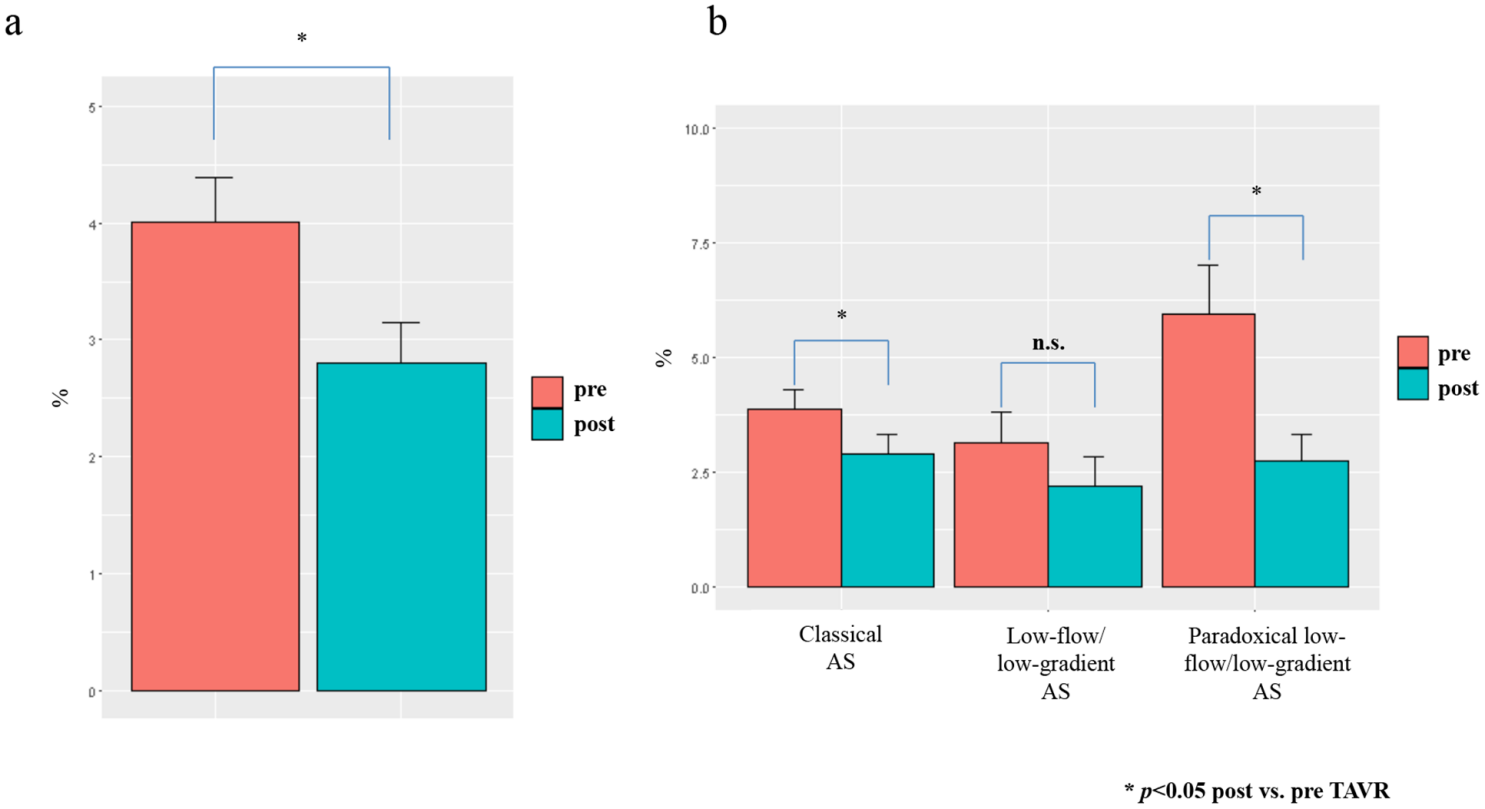
Decrease of intermediate monocytes after TAVR. Monocytes measured 4–7 days after TAVR-procedure by flow cytometry, (a) regarding all patients and (b) split up for different AS entities.

The percentage of intermediate CD14^++^CD16^+^ monocytes significantly declined early after TAVR procedure in the overall cohort of TAVR patients (Fig 1), while no significant differences in the absolute monocyte counts were found (Table 1). Taking a closer look at the different entities of aortic stenosis, the decline of intermediate CD14^++^CD16^+^ monocytes was significant in patients with classical as well as with paradoxical low-flow/low-gradient AS and showed a prominent, albeit insignificant trend in low-flow/flow-gradient AS (Fig 1).

We compared the intermediate monocyte counts from our TAVR-patient cohort to unselected patients hospitalized for different cardiovascular causes but not aortic stenosis representing a cross section of patients in our department, as well as to patients suffering from severe symptomatic mitral valve regurgitation (MR) at a comparable age undergoing percutaneous mitral valve repair (PMVR) with the MitraClip^®^ system (S2 Fig). Compared to most AS-patients pre TAVR the control group showed lower and MR-patients increased intermediate monocyte counts. However, PMVR was not followed by a decline in intermediate monocytes.

**Fig 2:**
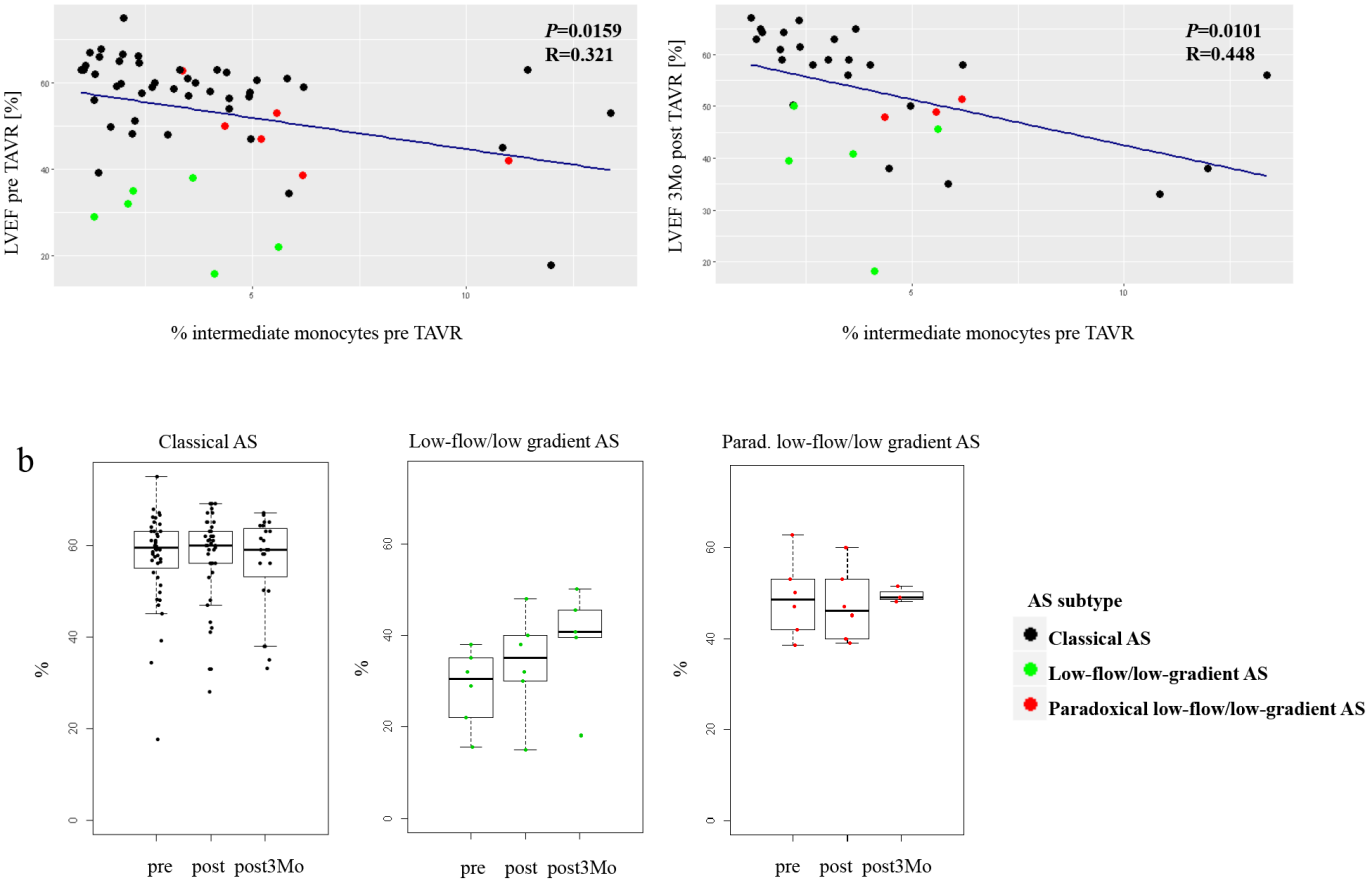
Intermediate monocytes and LVEF. (a) Correlation between intermediate monocytes measured by flow cytometry before TAVR-procedure and LVEF before and three months after TAVR-procedure. (b) Development of LVEF over time split up for different AS entities.

Intermediate monocytes pre TAVR correlated significantly with left ventricular function (LVEF) prior and at three months after TAVR (Fig 2). In patients presenting with low-flow/low-gradient aortic stenosis LVEF improved over time, while LVEF remained almost unchanged in classical and paradoxical low-flow/low-gradient AS patients (Fig 2).

**Fig 3:**
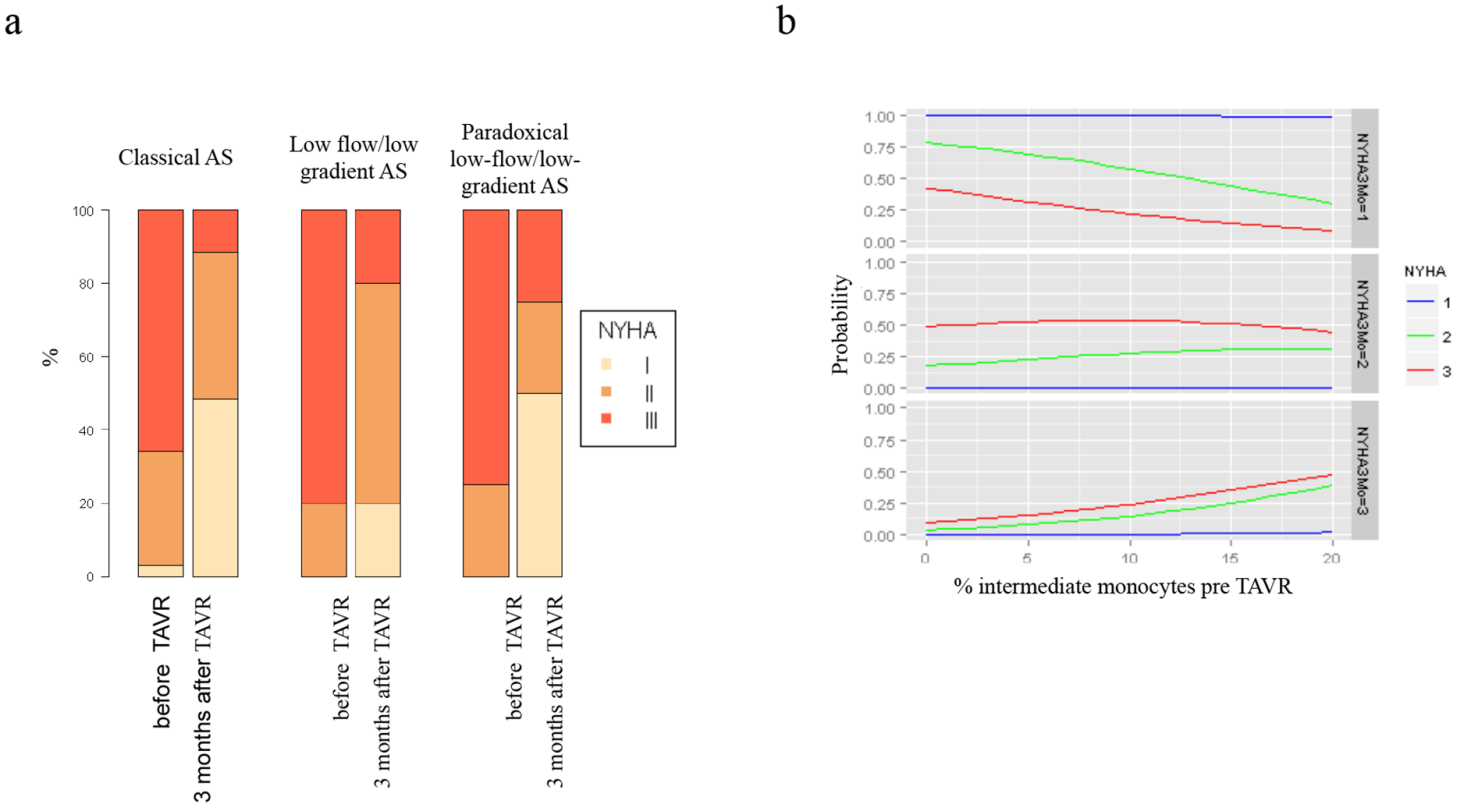
Functional capacities and intermediate monocytes. (a) Gain of functional capacities over time measured by NYHA class split up for different AS entities. (b) Predicted probabilities to reach a specific NYHA class after three months for different percentages of the variable intermediate monocyte determined before TAVR relative to class I chosen as baseline. NYHA classes before TAVR are depicted in blue: I; green: II; red: III.

Almost all patients gained functional capacity at three months post TAVR, when classified by New York Heart Association (NYHA) functional class (Fig 3). High amounts of intermediate monocytes prior TAVR were associated with worse functional outcome after 3 months and indicated a lower probability to reach NYHA I level (Fig 3).

**Fig 4:**
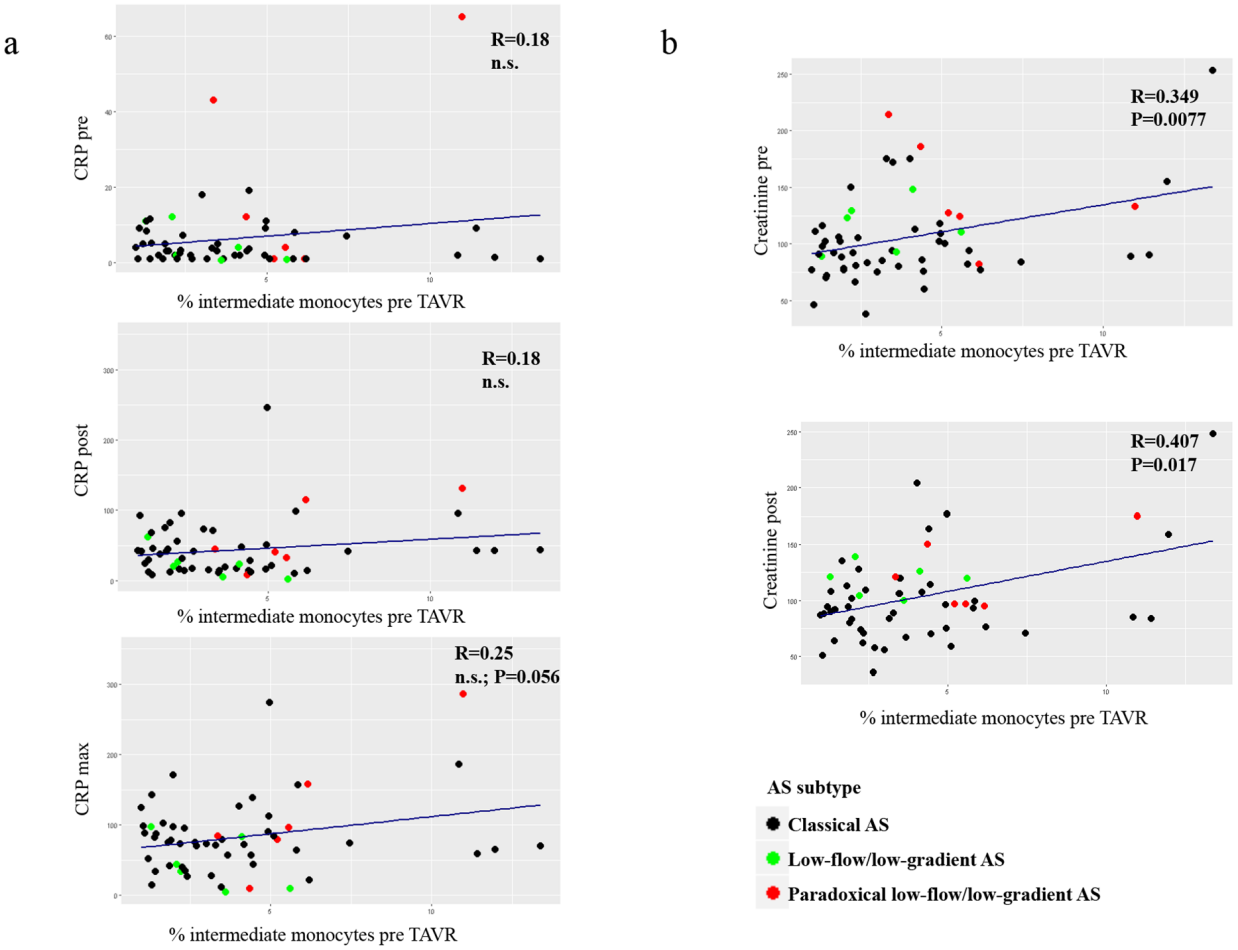
Correlations of intermediate monocytes with CRP and creatinine. (a) Correlations between intermediate monocytes measured by flow cytometry with serum CRP levels [mg/l] before and at day 4–7 after TAVR-procedure as well as intermediate monocytes before with maximum CRP-level between TAVR procedure and day 4–7. (b) Correlations between intermediate monocytes and creatinine-levels (μmol/l) before and early after TAVR-procedure.

**Fig 5:**
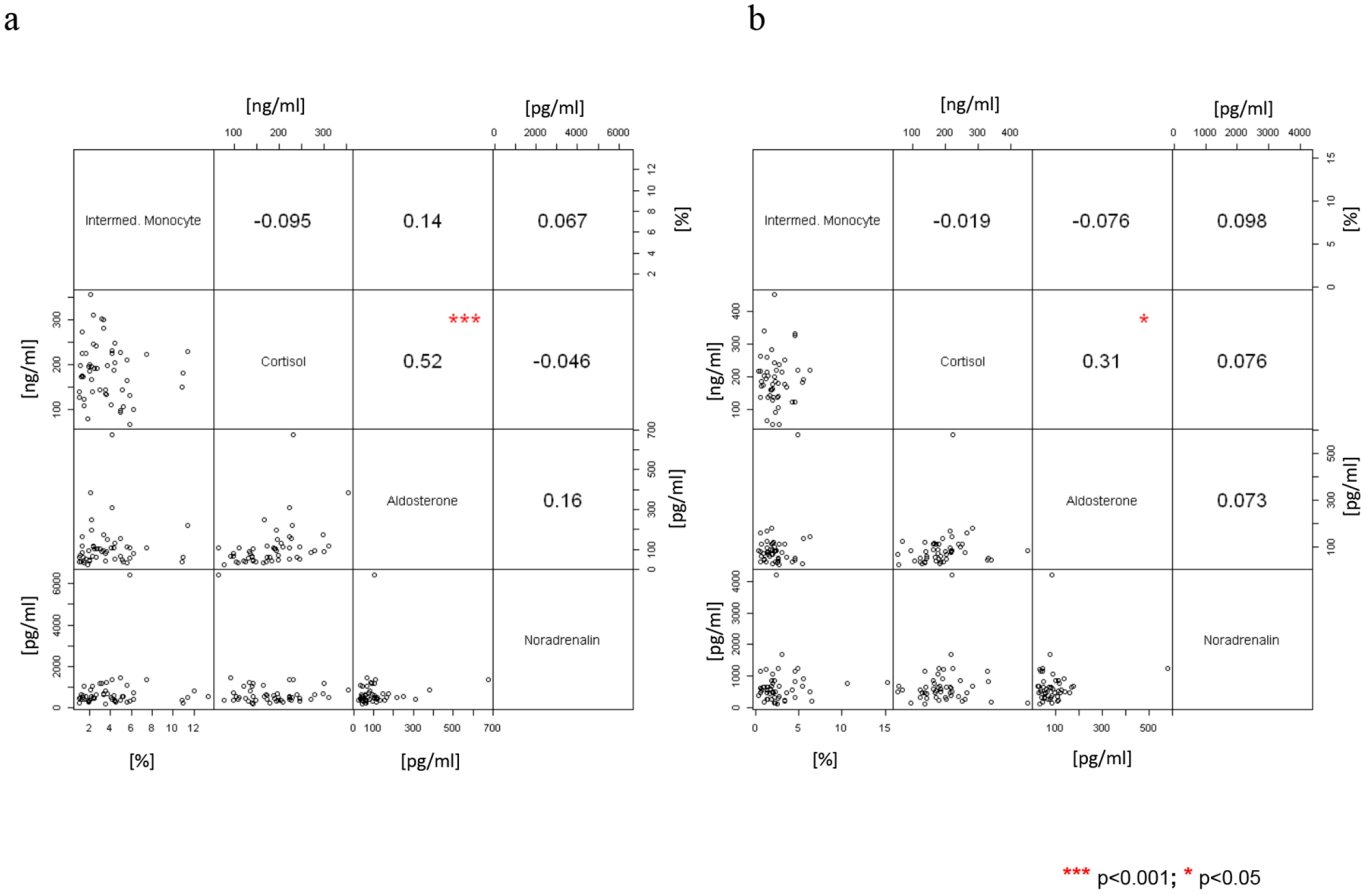
Correlations of intermediate monocytes with cortisol, aldosterone and noradrenaline. Correlations (Spearman) between intermediate monocyte measured by flow cytometry and cortisol (cor), aldosterone (aldo) as well as noradrenaline (nor). (a) Intermediate monocyte pre TAVR vs. cor, aldo, nor pre TAVR. (b) Intermediate monocyte post TAVR vs. cor, aldo, nor post TAVR. Correlations among cor, aldo and nor are also shown. On the top of each graph: pairwise correlation value and relative significance (negative value indicates inverse correlation); on bottom: bivariate scatterplot. ** p<0.01.

Intermediate monocytes did not correlate with clinical risk scores such as STS risk (R = 0,048, p = 0,7248) or EUROSCORE II (R = 0,011, p = 0,9344). Renal function measured by creatinine levels remained stable and correlated with intermediate monocytes (Table 2, Fig 4). C-reactive protein (CRP) levels as a marker of inflammation raised significantly after TAVR, but a significant correlation with intermediate monocytes could not be seen TAVR (Table 2, Fig 4). When looking at neurohomural activation we did not see any significant changes in serum levels of cortisol, aldosterone and noradrenalin after TAVR and values did not correlate with intermediate monocyte counts (Table 2, Fig 5).

## Discussion

To the best of your knowledge this is the first report investigating monocytes subsets in patients suffering from severe AS undergoing TAVR. Intermediate CD14^++^CD16^+^ monocytes did not only decline significantly after TAVR, but interestingly values before TAVR correlated with worse cardiac function and predicted probability for improvement in NYHA functional class at 3 months after TAVR.

Elevated intermediate monocyte counts are associated with various cardiovascular diseases and their prognostic value has been demonstrated before.[15–18, 24] However, understanding of monocyte subtype regulation remains mostly incomplete. LV function was shown to be an independent risk factor for mortality after surgical aortic valve replacement and we found the amount of intermediate monocytes before TAVR procedure to correlate with LVEF before and at three months after TAVR.[26] We further took a closer look at the monocyte subtype distribution in the different entities of AS, which are distinguished inter alia by left ventricular function. Intermediate monocyte cell count and their decline after resolving valve stenosis were not distributed equally among the heterogeneous cohort of patients. So variation in hemodynamic conditions within the heart may influence shifts in monocyte subsets. Besides these aspects it has been shown years ago that functional capacity and left ventricular ejection fraction correlate only weakly.[31–34] In our observational study intermediate monocytes though were able to predict functional capacity at 3 months after TAVR, potential predicting outcome independent from LVEF.

In critically ill patients undergoing cardiac valve surgery increased levels of CD14^+^CD16^+^ monocytes were shown.[22] To evaluate perioperative risk of patients undergoing heart surgery including aortic valve replacement, clinical scores such as STS risk score or EuroSCORE II are used and therefore represent the clinical condition of patients. Nevertheless, we did not see any correlation of intermediate monocytes levels before TAVR procedure with these widely accepted risk scores, so monocytes distribution seems to not just reflect patients’ over all clinical status.

Patients received DAPT following TAVR if no indication for anticoagulation was present, which might influence monocyte subset distribution. However, a similar therapeutical standard exists for patients suffering from severe MR undergoing PMVR and no decline in intermediate monocytes was observed in these patients. Furthermore a notable number of patients did not receive new DAPT because indication for anticoagulation was present or DAPT was already applied previous to TAVR-procedure. Other pre-existing medication was continued. We therefore do not suspect any relevant influence of medication in particular DAPT on monocyte subset distributions.

Monocyte subsets are shifted towards the intermediate monocyte subtype in inflammation and reports about elevated CRP-levels in patients with degenerative AS suggest a regulation of monocyte subset distribution by inflammation.[35, 36] Though, we saw an increase in CRP-levels early after valve stenosis was treated with TAVR, which can be explained by inflammation due to post-interventional healing processes at the vascular access sites and within the left ventricular outflow tract. CRP-levels however, did not correlate with the amount of intermediate monocytes TAVR at any point. Consequently inflammation seems to play an indecisive role in monocyte regulation in patients undergoing TAVR.

Epidemiological studies showed an increase of intermediate monocytes with worsening of renal function and new reports state a uremia-induced generation of intermediate monocytes by dysregulation of DNA methylation.[24, 37] We identified in our TAVR cohort a correlation of intermediate monocytes with creatinine levels before and shortly after TAVR. However, renal function did not vary significantly after TAVR compared to values before the procedure. Renal function therefore influences monocytes subset distribution, but apparently independent from AS status.

The innate immune system is affected by the sympathetic immune system as well.[27–29] Monocytes express adrenoceptors and i.e. stress due to physical exercise seems to alter adrenoceptor density on monocytes’ cell surface.[38] Adrenoceptor activation results in anti-inflammation and immunosuppression.[39–42] However, serum levels of noradrenaline did not correlate with intermediate monocyte subtype in TAVR-patients.

Corticosteroids possess various effects, as well on the innate immune system; for example glucocorticoid therapy has been associated with a shift towards intermediate monocytes before.[30] We investigated possible effects of endogenous corticosteroids on monocyte subset distribution. However, no significant correlation between serum cortisol levels and intermediate monocyte amount was observed. Monocyte mineralocorticoid receptor signaling was shown to regulate basal monocyte/macrophage function as well, so we assessed serum aldosterone levels and their association with intermediate monocyte cell counts, which again did not show any significant correlations.[43]

So various factors seem to influence monocyte subset distribution at distinct extends. However, the decrease in intermediate monocyte counts we found after resolving valve stenosis by TAVR procedure was not clearly related to any other alteration of possible influences we investigated. Most likely hemodynamic conditions within the heart may critically influence monocyte subset distribution.

### Study limitations

Further studies especially concerning the distributions of monocyte subsets in the different entities of severe AS will be needed to reveal their clinical relevance in this setting. Particularly the number of patients suffering from low-flow/low-gradient or paradoxical low-flow/low-gradient AS remained too small to make assumptions regarding the prognostic relevance of monocytes in these AS subpopulations. Moreover, even though monocyte mobilization has frequently been reported in various diseases and their prognostic value has been described repeatedly, the question, whether this represents a reflection or rather contributes to the diseases’ progression remains essentially unanswered. [16, 17, 44]

## Conclusions

Interestingly the amount of intermediate monocytes before TAVR negatively correlated with the probability to gain functional capacity expressed in NYHA functional class, offering a possible prognostic value of intermediate monocytes for patients suffering from AS undergoing TAVR procedure. Therefore, assessment of intermediate monocytes might add information for an enhanced selection of AS-patients with highest probability to profit best from TAVR.

## Supporting information

S1 Fig **a Gating strategy:** After pre-selection in side scatter (SSC) vs. forward scatter (FSC) dot plot and FSC vs. Time-of-Flight (ToF) dot plot, monocytes were identified as HLA DR^+^ cells and further stratified by CD14/CD16 expression. CD14^++^/CD16^−^ classical, CD14^++^/CD16^+^ intermediate, CD14^+^/CD16^++^ non-classical monocytes. **b Example of Monocytes Distribution pre/post TAVR**.
(TIF)

S2 Fig Comparison of intermediate monocyte (%) in different patient cohorts displayed as a dot plot graph. Values pre TAVR (red, dark green, green) respectively PMVR (*MitraClip*, violet) are shown on the x-axis, while values post TAVR respectively PMVR are shown on the y-axis. Control aged-patients are displayed separately in same orientation as values pre intervention, since no intervention was performed (blue). Large points represent centroids of subsets with se.
(TIF)

S1 Table Results of Shapiro-Wilk test to verify normality. ASTyp = Aortic Stenosis Type, 1 Classical; 2 Low flow/low gradient; 3 Paradoxical Low flow/low gradient.
(DOCX)
