# Supplementary figures and images for: Intermediate CD14++CD16+ monocytes decline after transcatheter aortic valve replacement and correlate with functional capacity and left ventricular systolic function
Source: PLoS One. 2017 Aug 22;12(8):e0183670. doi: 10.1371/journal.pone.0183670 (PMC5568735; doi:10.1371/journal.pone.0183670)

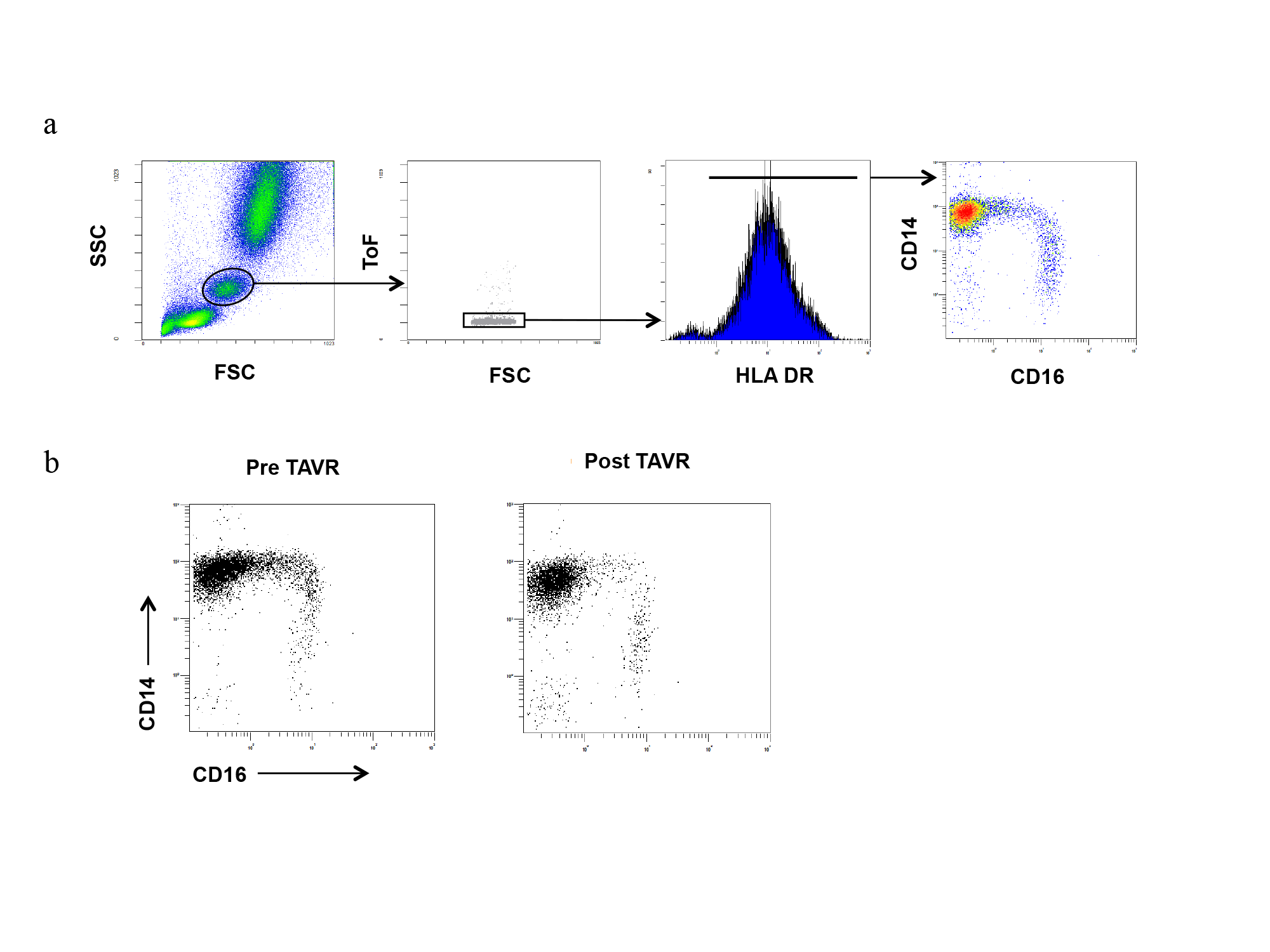

Supplement: S1 Fig — a Gating strategy: After pre-selection in side scatter (SSC) vs. forward scatter (FSC) dot plot and FSC vs. Time-of-Flight (ToF) dot plot, monocytes were identified as HLA DR+ cells and further stratified by CD14/CD16 expression. CD14++/CD16− classical, CD14++/CD16+ intermediate, CD14+/CD16++ non-classical monocytes. b Example of Monocytes Distribution pre/post TAVR. (TIF) [file pone.0183670.s001.tif]

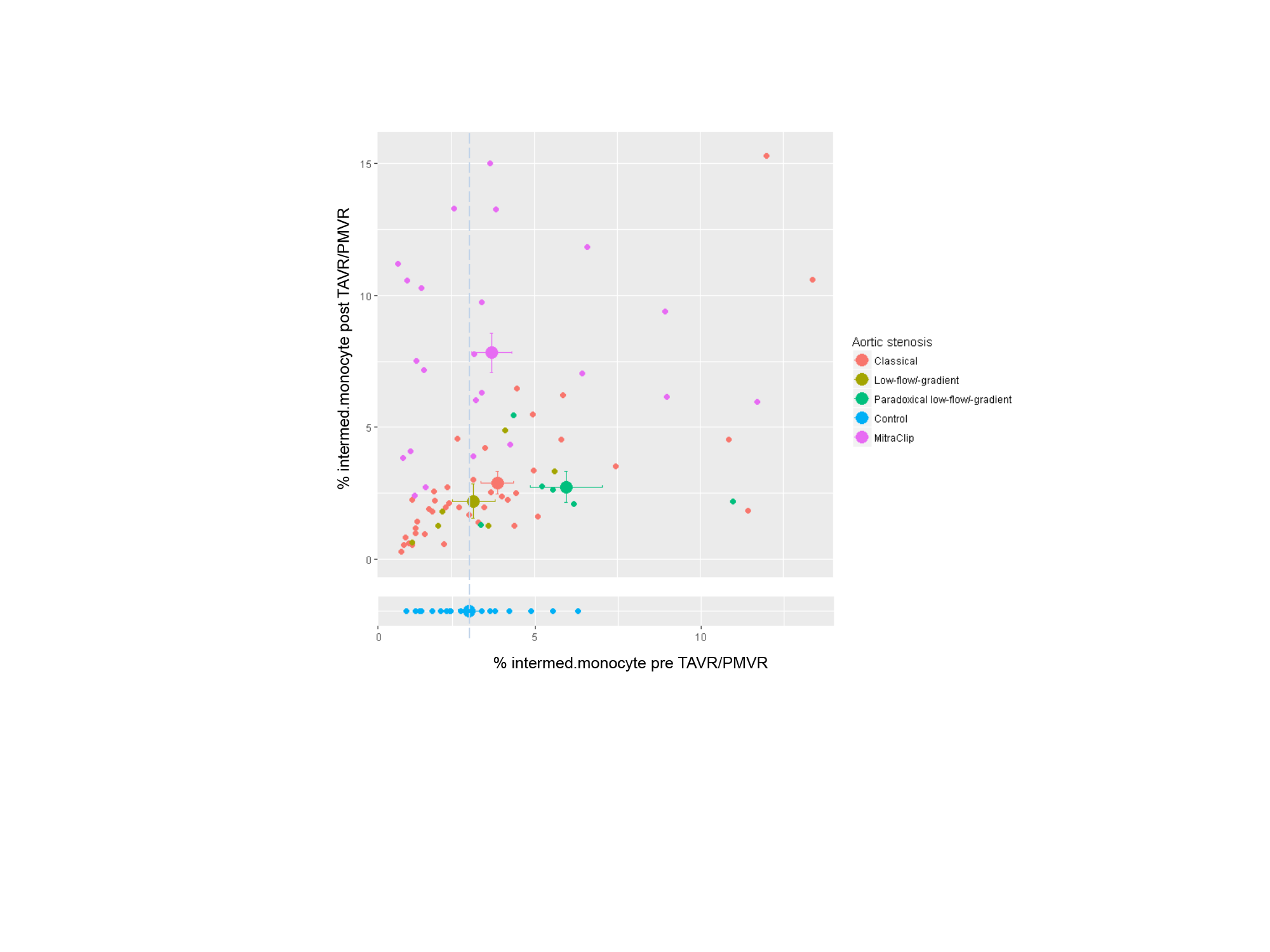

Supplement: S2 Fig — Values pre TAVR (red, dark green, green) respectively PMVR (MitraClip, violet) are shown on the x-axis, while values post TAVR respectively PMVR are shown on the y-axis. Control aged-patients are displayed separately in same orientation as values pre intervention, since no intervention was performed (blue). Large points represent centroids of subsets with se. (TIF) [file pone.0183670.s002.tif]
