# Supplementary material for: Intermediate CD14++CD16+ monocytes decline after transcatheter aortic valve replacement and correlate with functional capacity and left ventricular systolic function
Source: PLoS One. 2017 Aug 22;12(8):e0183670. doi: 10.1371/journal.pone.0183670 (PMC5568735; doi:10.1371/journal.pone.0183670)
Supplement: S1 Table — ASTyp = Aortic Stenosis Type, 1 Classical; 2 Low flow/low gradient; 3 Paradoxical Low flow/low gradient. (DOCX) [file pone.0183670.s003.docx]

**Shapiro-Wilk-Test**

**Variable Shapiro-Wilk test P-value**

Interm. Monocyte pre TAVR [%] not normal 7.668e-07

Interm. Monocyte post TAVR [%] not normal 7.537e-09

Classical Monocyte pre TAVR [%] not normal 0.04085

Classical Monocyte pre TAVR [%] not normal 0.004323

Non classical Monocyte pre TAVR [%] not normal 0.002213

Non classical Monocyte post TAVR [%] not normal 0.0005128

Interm. Monocyte pre TAVR [n/µL] not normal 1.007e-07

Interm. Monocyte post TAVR [n/µL] not normal 3.547e-09

Classical Monocyte pre TAVR [n/µL] normal 0.5011

Classical Monocyte pre TAVR [n/µL] normal 0.2234

Non classical Monocyte pre TAVR [n/µL] not normal 4.148e-05

Non classical Monocyte pre TAVR [n/µL] not normal 0.00315

Interm. Monocyte ASTyp 1 [%] not normal 1.686e-06

Interm. Monocyte ASTyp 2 [%] normal 0.7504

Interm. Monocyte ASTyp 3 [%] normal 0.1242

LVEF pre TAVR [%] not normal 6.018e-05

LVEF post TAVR [%] not normal 0.000106

LVEF 3Mo post TAVR [%] not normal 0.008666

LVEF pre TAVR ASTyp 1 [%] not normal 3.356e-05

LVEF post TAVR ASTyp 1 [%] not normal 2.732e-05

LVEF 3Mo ASTyp 1 [%] not normal 0.0007017

LVEF pre TAVR ASTyp 2 [%] normal 0.7331

LVEF post TAVR ASTyp 2 [%] normal 0.8105

LVEF 3Mo ASTyp 2 [%] normal 0.198

LVEF pre TAVR ASTyp 3 [%] normal 0.9082

LVEF post TAVR ASTyp 3 [%] normal 0.6148

LVEF 3Mo ASTyp 3 [%] normal 0.5542

CRP pre TAVR [mg/l] not normal 1.798e-12

CRP post TAVR [mg/l] not normal 3.019e-08

CRP max [mg/l] not normal 1.446e-05

Creatinine pre TAVR not normal 2.928e-05

Creatinine post TAVR not normal 0.0002208

AV Gradient max pre TAVR [mmHg] normal 0.1314

AV Gradient max post TAVR [mmHg] not normal 5.015e-06

AV Gradient max 3Mo [mmHg] not normal 1.903e-05

AV Gradient mean pre TAVR [mmHg] normal 0.05199

AV Gradient mean post TAVR [mmHg] not normal 9.188e-08

AV Gradient mean 3Mo [mmHg] not normal 4.449e-12

AV VTI pre TAVR [cm] normal 0.6505

AV VTI post TAVR [cm] not normal 0.0005221

AV VTI 3Mo [cm] not normal 0.008563

Cortisol pre TAVR [ng/mL] normal 0.5039

Cortisol post TAVR [ng/mL] not normal 0.01503

Aldosterone pre TAVR [pg/mL] not normal 3.937e-10

Aldosterone post TAVR [pg/mL] not normal 2.416e-11

**Variable Shapiro-Wilk test P-value**

Noradrenalin pre TAVR [pg/mL] not normal 2.153e-13

Noradrenalin post TAVR [pg/mL] not normal 1.213e-10

Leukocytes pre TAVR [n/µL] normal 0.5509

Leukocytes post TAVR [n/µL] normal 0.21

Neutrophiles pre TAVR [n/µL] normal 0.05926

Neutrophiles post TAVR [n/µL] not normal 0.01451

Lymphocyteses pre TAVR [n/µL] not normal 0.003218

Lymphocyteses post TAVR [n/µL] normal 0.3285

Monocytes pre TAVR [n/µL] normal 0.7051

Monocytes post TAVR [n/µL] normal 0.318
